# Identification of Salmonella enterica biovars Gallinarum and Pullorum and their antibiotic resistance pattern in integrated crop-livestock farms and poultry meats

**DOI:** 10.1099/acmi.0.000775.v6

**Published:** 2024-09-30

**Authors:** Dita Julianingsih, Zabdiel Alvarado-Martinez, Zajeba Tabashsum, Chuan-Wei Tung, Arpita Aditya, Sarika Kapadia, Saloni Maskey, Aditi Mohapatra, Debabrata Biswas

**Affiliations:** 1 Department of Animal and Avian Sciences, University of Maryland, College Park, MD 20742, USA; 2 Biological Sciences Program, University of Maryland, College Park, MD 20742, USA

**Keywords:** antibiotic resistance, gallinarum, pasture farms, prevalence, pullorum, *Salmonella enterica*

## Abstract

Due to consumer demand, many conventional poultry farms are now growing poultry without antibiotics or synthetic chemicals. In addition to this, pasture/organic poultry farms have increased significantly in the USA, and they are also antibiotic- and chemical-free. According to recent reports, both antibiotic-free conventional and pasture poultry farmers are facing the re-emergence of bacterial diseases. Bacterial diseases cause higher mortality rates in birds and lead to non-profitable poultry farming. This study investigated the prevalence of *Salmonella enterica* subsp. *enterica* serovar Gallinarum biovars Gallinarum (*S*. Gallinarum), the causative agent of fowl typhoid, and *Salmonella enterica* subsp. *enterica* serovar Gallinarum biovars Pullorum (*S*. Pullorum), the causative agent of pullorum disease, within integrated crop-livestock/pasture farm environments and their processed products. Specifically, the study focused on both the pre-harvest period, which includes the conditions and practices on the farm before the crops and livestock are harvested, and the post-harvest period, which encompasses the handling, processing, and storage of the products after harvest. A total of 1286 samples were collected from six farms and adjacent 13 markets to determine the prevalence of *S*. Gallinarum and *S*. Pullorum by using both microbiological culture and molecular techniques, specifically PCR. Antimicrobial susceptibility testing was performed using the agar dilution method for the recommended antibiotics as described in the Clinical Laboratory Standards Institute (CLSI). *S*. Pullorum was detected in 11 samples (2.7%), while *S*. Gallinarum was found in six samples (1.5%) out of a total of 403 samples at the pre-harvest level. At the post-harvest level, only *S*. Gallinarum was identified in 14 meat samples out of 883(1.6%) recovered from samples collected from retail markets. Antibiogram showed *S*. Gallinarum and *S*. Pullorum to be highly resistant to cephradine, trimethoprim-sulfamethoxazole, amoxicillin, streptomycin, and ampicillin. This data demonstrates that both *S*. Pullorum and *S*. Gallinarum are commonly present in farm poultry environments as well as the products sold in the markets, which warrants implementation of regular surveillance and monitoring programmes, as well as potentially requiring future control strategies to reduce * S*. Pullorum and *S*. Gallinarum transmission.

**Impact Statement**

This study reveals a notable re-emergence of bacterial diseases in US poultry farms due to the widespread adoption of antibiotic-free practices. Focusing on *S*. Pullorum and *S*. Gallinarum, causative agents of fowl typhoid and pullorum disease, the research demonstrates their prevalence in farm poultry environments and market products. High antibiotic resistance poses a serious threat, emphasizing the need for immediate surveillance and control strategies. This work significantly contributes to the literature, informing policies and practices to safeguard both poultry and consumers.

## Data Summary

All data associated with this work is reported in the article and supplement.

## Introduction

Poultry meat is one of the most popular animal-source proteins consumed worldwide, spanning a wide range of countries, customs, and religions [1]. Global imports of poultry products rose 4% a year on average from 2001 to 2021 as the market’s demand climbed [2]. In the next 10 years, poultry is anticipated to continue being the most widely imported livestock product by volume [3]. The notable surge in the value of poultry production and sales in the US, amounting to $46.1 billion in 2021 and $35.1 billion from the previous year, highlighting the remarkable economic advantages of chicken farming [4]. Compared to other livestock, chickens mature and gain weight faster, converting feed to meat more effectively. For farmers in developing nations and emerging markets, chicken farming is easier and more affordable than beef and pork [2]. To address the growing consumer demand, several poultry producers, particularly those on small- or mid-scale diversified farms, such as integrated crop-livestock and backyard farms, are choosing to raise birds in pasture or free-range systems that allow poultry to be raised in less constrained conditions with access to the outdoors [5,6]. However, free-range poultry production, such as backyard and integrated crop-livestock farms (with poultry component), which allow for outdoor access, can potentially elevate the incidence of some diseases, including *Salmonella* infection from contact with infected birds, wild animals, and other vectors [7,8].

*Salmonella enterica* (SE) is an enteric pathogen that can infect both humans and animals [8,9]. The two most common avian pathogenic SE are *Salmonella enterica* subspecies *enterica* serovar Gallinarum biovar Gallinarum (*S*. Gallinarum), which causes fowl typhoid and results in significant death rates in poultry of all ages, and *Salmonella enterica* subspecies *enterica* serovar Gallinarum biovar Pullorum (*S*. Pullorum), which causes pullorum disease with very high mortality (potentially approaching 100%) in young chickens and turkeys within the first 2–3 weeks of age [10]. It is often followed by chronic infection in adults by vertical transmission [11,12]. These bacterial diseases in conventional poultry were under control in the US but sporadic cases were reported in free-range systems, such backyard and integrated crop-livestock farms [13,15].

In recent years, there has been an alarming rise in the prevalence of these bacterial pathogens in both antibiotic-free conventional and free-range poultry farming [16]. Furthermore, the increasing number of antimicrobial resistances in SE serovars is a significant concern in both veterinary and human medical settings. This resistance complicates treatment options, leading to challenges in managing infections effectively [17,19]. The isolates of *S*. Gallinarum showed frequent resistance to oxytetracycline, doxycycline, amoxicillin, sulfamethoxazole, enrofloxacin, and neomycin [20]. According to a study by Sun *et al.* [21], generally, 80.5%* S*. Pullorum isolates were resistant to at least one antibiotic. In total, 42% isolates of the *S*. Pullorum bacterium were resistant to three or more classes of antibiotics, with ampicillin-tetracycline-nalidixic acid (13.6%) being the most prevalent pattern. As such, addressing antibiotic resistance in *Salmonella* becomes crucial for both animal welfare and human health.

Therefore, this study aims to investigate the prevalence of *S*. Gallinarum and *S*. Pullorum in the poultry environment at the farm level from integrated crop-livestock farms (ICLFs) and post-harvest chicken meats from farmers markets, organic retailers, and conventional retailers. Additionally, this study seeks to determine the antimicrobial resistance patterns of *S*. Gallinarum and * S*. Pullorum at both pre-harvest and post-harvest levels. This study aims to contribute to the creation of efficient monitoring and control strategies to inform laws that protect the poultry industry and public health by shedding light on the scope and effects of *S*. Gallinarum and *S*. Pullorum infections and antibiotic resistance in poultry.

## Methods

### Sample types and collection

A combined total of 1 286 samples were collected from pre-harvest and post-harvest levels. At the pre-harvest level, 403 samples were collected from seven ICLFs in the Maryland-Washington DC area during the summer periods of 2019 to 2021. These farms practice pasture systems and refrain from using subtherapeutic antibiotics or synthetic chemicals, including pesticides, herbicides, and fertilizers. The environmental samples collected from these farms include water, grass, bedding, feed, compost, and soil are shown in Table 1. At the post-harvest level, a total of 883 processed chicken meat samples were collected by Peng *et al.* [8] in a previous study conducted from 2012 to 2014. These samples were obtained from seven farmer markets, three organic retail supermarkets, and three conventional retail supermarkets (Table 2). All samples were then microbiologically analysed to assess the prevalence of *S*. Pullorum and *S*. Gallinarum at the post-harvest level.

**Table 1.** Pre-harvest samples across different poultry environment categories collected from farms in 2019–2021

Sample sources	Sample description	Sample no.
Poultry water	Water from the trough in the chicken and turkey housing facility	80
Poultry grass	Plant materials, weeds, and grass from pasture grounds and the surrounding area	55
Poultry bedding	Fresh and used bedding materials from chicken and turkey housing facility	41
Poultry soil	Soil and dirt from chicken and turkey pasture grounds	57
Poultry feed	Fresh and partially eaten feeding material in chicken and turkey housing facility	76
Poultry compost	Organic material from the surface and inner layers of the compost pile	5
Poultry faeces	Fresh and old chicken and turkey droppings from pasture and housing facility	89
Total sample		403

**Table 2.** Post-harvest/meat samples across different categories collected from 2012 to 2014 [8]

Sample sources	Sample description	Sample no.
Organic retail chicken (ORC)	Whole chicken and partial chicken meat	237
Farmers market chicken (FMC)	Whole chicken and partial chicken meat	252
Conventional retailer chicken (CRC)	Whole chicken and partial chicken meat	394
Total sample		883

### Microbiological analysis for *Salmonella* detection

Samples from farm environments were collected using sterile equipment, such as swabs or containers. These samples were handled aseptically to prevent contamination, then transported to the lab under controlled conditions, typically on ice packs or in insulated containers, to maintain microbiological integrity. Meat samples were sampled aseptically at various markets. These samples were carefully packaged and transported to the lab following established protocols to preserve sample quality and prevent bacterial growth during transit. Each type of sample was handled individually in accordance with Bacteriological Analytical Manual [22]. Processing and enrichment of samples for later bacterial isolation were performed as described in a previous study [8]. Briefly, 1 g of faeces, 3 g of soil, compost, grass, bedding, and feed samples contained in Whirl-Pak bags were suspended in 9 ml of Luria–Bertani (LB) broth (AMRESCO, USA) supplemented with a 10% Sheep blood (Ward’s Science, USA) and incubated for 24 h at 37 °C. Meat products were separated into 25 g samples that were properly rinsed with 0.1% Buffered Peptone Water (BPW) (Himedia, India). Subsequently, 10 ml of the rinse solution was combined with an equal volume of double-concentrated LB supplemented with 10% sheep blood and incubated for 24 h at 37 °C [8]. After enrichment, a 10 µl of LB broth was streaked on a xylose lysine deoxycholate (XLD) agar plate, which was incubated for 24 h at 37 °C [10,23]. Then, typical colonies were selected from the XLD agar and subcultured on LB agar and later used to prepare stocks in LB broth with 20% glycerol that were stored at −80 °C.

## Confirmation of *S. Gallinarum* or *S. Pullorum*

PCR were employed to validate the culturally identified potential *Salmonella* isolates [24, 25]. Using the DNeasy Blood and Tissue Kit (Qiagen, USA), genomic DNA from isolates was isolated from overnight cultures in accordance with the manufacturer’s instructions. The primers used for the PCR used in this study (Table 3) were bought from Thermo Fisher Scientific (USA). To verify the specificity of primers used, the PCR reactions with negative controls, specifically using water, PCR reagent (no DNA), and DNA of a highly related *Salmonella* serovar/serotype were used (shown in the Supplementary Material, available in the online version of this article). To identify the isolates as SE species, preliminary *SalOriC* and *aceK* PCR was conducted. *SalOriC* and *aceK* gene were used because they are highly conserved within the *Salmonella enterica* species, providing a specific and reliable marker for the identification of this pathogen. The standard strain of *S*. Pullorum ATCC 13036 and *S*. Gallinarum CAT375 from Presque Isle Cultures, Erie, PA, were utilized as the positive controls in this study. The PCR assay based on the *ratA* gene was used to differentiate *S*. Gallinarum from *S*. Pullorum due to distinct sequence variations between these two biovars. Specifically, the product size for *S*. Pullorum is 243 bp, while for *S*. Gallinarum, it is 1047 bp, allowing for accurate differentiation of the isolates (Table 3) [12,26]. The reaction solution for the PCR assays contained one buffer with KCl, 160 µM of each deoxynucleotide triphosphate, 1.5 mM of magnesium chloride, 0.6 µM of each primer, 0.75 U of Taq DNA polymerase, 1 µl of DNA template (at least 10 ng µl^–1^), and up to 20 µl of ultra-pure water. Initial denaturation took place at 94 °C for 3 min, then there were 25 cycles of 94 °C for 1 min, 63 °C for 30 s, and 72 °C for 60 s, with a final step of 72 °C for 5 min. The following electrophoresis at 4 V cm^–1^ for 60 min on a 1.5% (w/v) agarose gel stained with ethidium bromide, imaging was used to examine the PCR data [12].

**Table 3.** Primers utilized in this investigation to identify and subtype *Salmonella* species

Primer name	Primer sequences (5’−3’)	Product sizes (bp)	References
*aceK*-F	CCGCGCTGGTTGAGTGG	240	O’ Regan *et al*. [24]
*aceK*-R	GCGGGGCGAATTTGTCTTTA		
*SalOriC*-F	GCGGTGGATTCTACTCAAC	461	Woods *et al*. [25]
*SalOriC*-R	AGAAGCGGAACTGAAAGGC		
*ratA*-F	GACGTCGCTGCCGTCGTACC	Pullorum: 243	Batista *et al.* [12]
*ratA*-R	TACAGCGAACATGCGGGCGG	Gallinarum: 1047	

### Determination of antimicrobial resistance pattern of confirmed *S.* Gallinarum and *S.* Pullorum

Both confirmed *S*. Gallinarum and *S*. Pullorum were evaluated for their antibiotic resistance pattern using a standard agar dilution method in accordance with the Clinical and Laboratory Standards Institute [27]. Antibiotic‐containing agar plates were created using three different breakpoint concentrations of the chosen antibiotics in molten Muller–Hinton (MH) agar (Becton Dickinson and Co) (Table 4) [8]. Isolates were cultured overnight on MH agar plates at 37℃ before inoculating to antibiotic-containing MH agar plates. For culturing each of the isolates, a single colony was selected and suspended in 1×PBS and adjusted to the McFarland Standard of 0.5 (OD_600_). Then, 2 µl of each bacterial culture (approximately 10^5^ c.f.u.) was inoculated on an antimicrobial-containing MH agar plate and incubated at 37℃ overnight. The MIC was defined as the lowest concentration of an antimicrobial agent that completely prevented an isolated bacterial strain from exhibiting observable growth. The results were analysed in line with CLSI [27] breakpoints (Table 4). Since these isolates can be therapeutically treated with a greater than usual dosage of the antibiotic, isolates with intermediate resistance were categorized as susceptible [8].

**Table 4.** Antibiotics tested in this study and their breakpoints*

Antibiotic category	Antibiotic name	Breakpoint concentrations (μg ml^–1^)		
		Susceptible	Intermediate	Resistant
Penicillin	Ampicillin	≥8	≥16	≥32
	Amoxicillin	≥8	≥16	≥32
Macrolide	Azithromycin	≥16	≥24	≥32
Cephalosporin	Cephradine	≥4	≥8	≥16
	Ceftriaxone	≥1	≥2	≥4
Phenolic	Chloramphenicol	≥8	≥16	≥32
Quinolone	Ciprofloxacin	≥0.06	≥0.12	≥1
Aminoglycoside	Gentamycin	≥4	≥8	≥16
	Kanamycin	≥16	≥32	≥64
	Streptomycin	≥16	≥24	≥32
Tetracycline	Tetracycline	≥4	≥8	≥16
	Oxytetracycline	≥4	≥8	≥16
Folate pathway inhibitors	Trimethoprim-sulfamethoxazole	≥2 and ≥38	≥3 and ≥57	≥4 and ≥76

*The standards outlined in the CLSI manual were used to determine the antibiotic breakpoints.

### Statistical analysis

Data analysis was performed using SAS version 9.3 (SAS Institute, USA) and involved Chi-square statistical analysis to determine the significant prevalence of SE, *S*. Gallinarum and *S*. Pullorum in various sample categories. This approach also investigated antibiotic resistance patterns of *S*. Pullorum and *S*. Gallinarum and potential connections in detecting these organisms within confirmed samples.

## Results

### Prevalence of SE in farm ecology and post-harvest chicken meat

Before delving into the assessment of *S*. Gallinarum and *S*. Pullorum prevalence, it is crucial to establish the prevalence of SE. Table 5 presents the prevalence of SE in the samples collected from diverse farm poultry environments. After molecular confirmation, it was determined that 60 out of 403 samples tested positive for SE, resulting in a prevalence rate of 14.9%. This data was subsequently analysed using a contingency table and a Chi-square test, which demonstrated that the results were statistically significant (*P*<0.05), indicating that the findings were not due to chance within the environmental samples. Contamination with SE was commonly found in all types of environmental samples (grass, soil, feed, faeces, bedding, and water) from Table 1, but the highest percentage (31.6%, 18/57) of SE was detected in collected poultry soil, indicating the soil’s potential role as a reservoir for the pathogen. The lowest contamination was in feed (2.33%, 2/76), suggesting that it may play a lesser role in the transmission and persistence of *Salmonella* in the farm poultry environment. It was also observed that SE was also highly prevalent in other sources that come into direct contact with poultry such as water (6.25%, 5/80), bedding (17%, 7/41), grass (12.7%, 7/55), and faeces (23.6%, 21/89), while compost samples had zero contamination. On the other hand, according to a previous study conducted by Peng *et al.* [8], the average prevalence of SE in chicken meat samples (Table 6) collected from retail stores and farmers’ markets was 17.9%(158/883) (*P*<0.05). At the post-harvest level, the prevalence of SE was the highest (30.95%, 78/252) in farmers' market chicken meat, followed by organic retail chicken samples (19.8%, 47/237) and conventional retail chicken samples (8.4%, 33/394).

**Table 5.** The 2019–2021 farm environmental samples at pre-harvest level

Sources	SE positive
Poultry soil	18/57
Poultry feed	2/76
Poultry faeces	21/57
Poultry water	5/80
Poultry grass	7/55
Poultry bedding	7/41
poultry compost	0/5
Total sample	60/403

**Table 6.** The 2012–2014 processed chicken meats at post-harvest level [8]

Sources	SE positive
Organic retail chicken (ORC)	47/237
Farmers' market chicken (FMC)	78/252
Conventional retail chicken (CRC)	33/394
Total	158/883

### Prevalence of *S.* Gallinarum and *S.* Pullorum in the farm environment and poultry meat

**Fig. 1.**
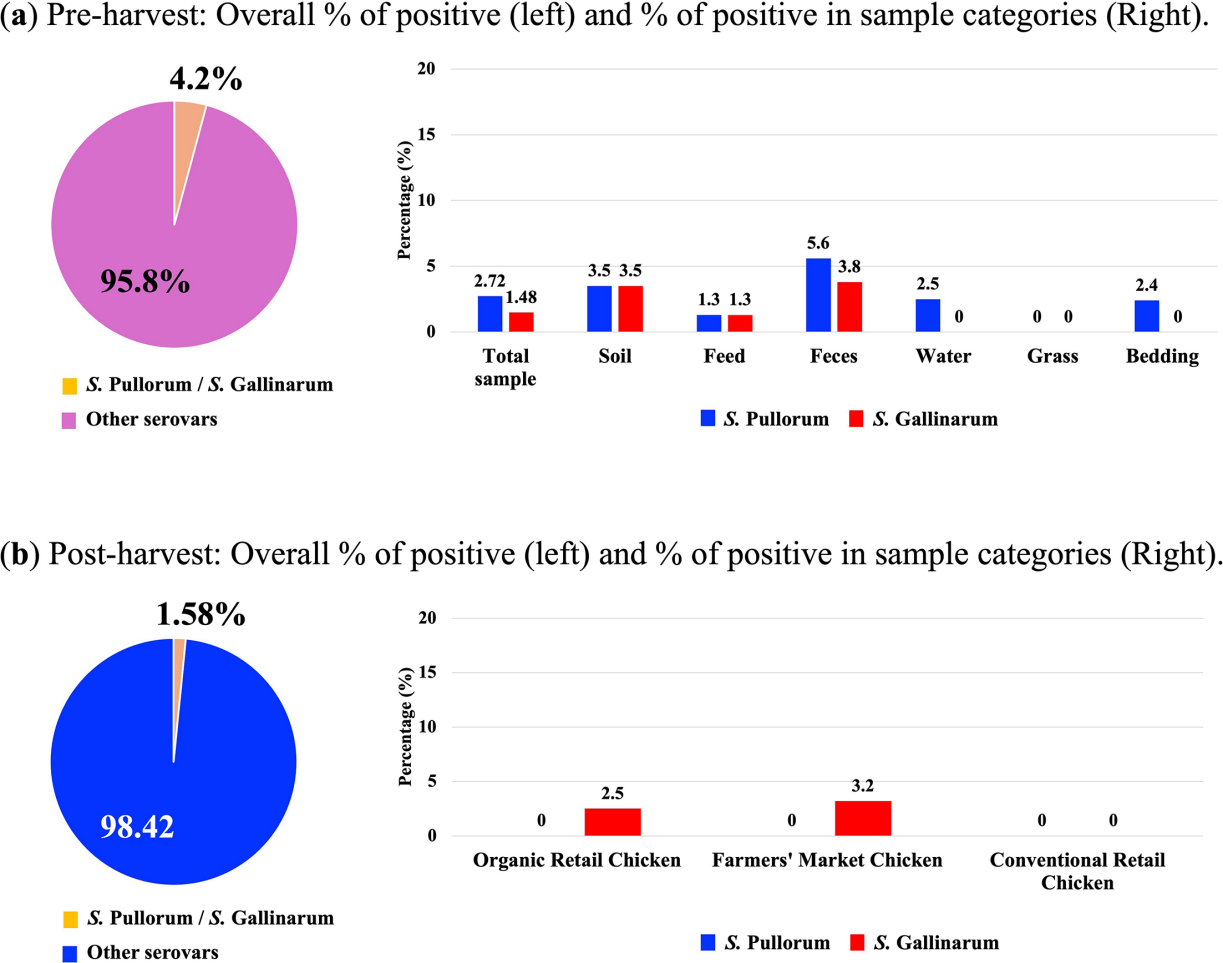
Prevalence of *S*. Gallinarum and *S*. Pullorum in poultry environment and product. (a) Pre-harvest, (b) Post-harvest. *S.* Pullorum and *S.* Gallinarum ecological distribution in samples collected from several markets and farms (Chi-square test, *p*<0.05) (a) Prevalence of *S.* Pullorum and *S.* Gallinarum in pre-harvest level (farm environments). (b) Prevalence of *S.* Pullorum and *S.* Gallinarum in post-harvest level (poultry meat samples from retail/market).

As a result of confirmed SE-containing samples, this study reveals the outcomes of a noticeable prevalence of *S*. Gallinarum and *S*. Pullorum in both poultry farm environments and post-harvest chicken meat products. As illustrated in Fig. 1a, among the different serovars of SE found in the samples collected from various farm poultry environments, 2.72%(11/403) were identified as *S*. Pullorum, and 1.48%(6/403) were identified as *S*. Gallinarum (4.2% combined) (*P*<0.05) and these serovars were not reported in our previous literature. These serovars found all types of environmental samples (grass, soil, feed, faeces, bedding, and water), with the highest prevalence (5.6%, 5/89) of *S*. Pullorum being detected in poultry faeces. In contrast, both grass and compost showed no contamination. It was also observed that *S*. Pullorum was regularly detected in other sources that commonly come into direct contact with poultry, such as water (2.5%, 2/80), soil (3.5%, 2/57), as well as feed (1.3%, 1/76) and bedding (2.4%, 1/41). Additionally, *S*. Gallinarum was found in faeces' samples (3.8%, 3/89), soil (3.5%, 2/57), and feed (1.3%, 1/76). However, * S*. Gallinarum was not detected in water, grass, or bedding samples. In contrast, the average prevalence of *S*. Gallinarum in chicken meat samples collected from retail stores and farmers’ markets was 1.6%(14/883), divided into the organic retail chicken (2.5%, 6/237) and farmers' market chicken (3.2%, 8/252) (Fig. 1b). *S*. Gallinarum was not detected in the conventional retail samples, and there was no sign of *S*. Pullorum in any meat samples.

## Antibiotic resistance patterns of isolated *S.* Gallinarum and *S.* Pullorum

**Fig. 2.**
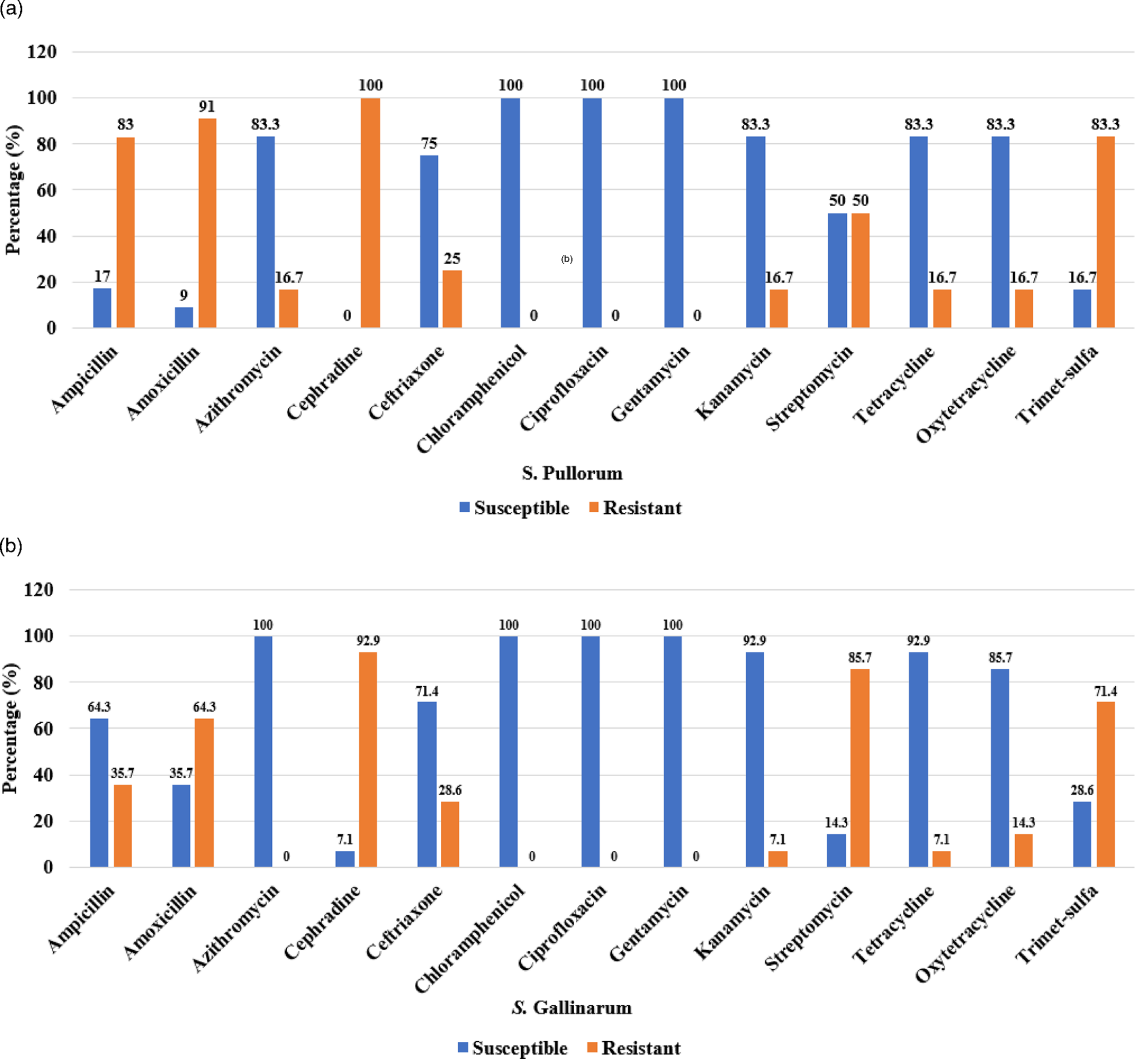
Antibiotic resistance pattern of *S.* Pullorum and *S*. Gallinarum. *S*. Gallinarum antibiotic resistance pattern. Antibiotic resistance profile of major *S.* Gallinarum and *S.* Pullorum isolated from various sources (Chi-square test, *p* < 0.05). (a) *S.* Gallinarum antibiotic resistance pattern. (b) *S.* Pullorum antibiotic resistance pattern.

A total of 20* S*. Gallinarum and 11* S*. Pullorum positive isolates from environmental and chicken meat samples from a previous study conducted by Peng *et al.* [8] were tested against 13 different antibiotics to determine their antibiotic resistance patterns (Fig. 2a). All *S*. Gallinarum isolates Fig. 2a) found in both farm poultry environments and meat samples were sensitive (100%) to azithromycin, chloramphenicol, ciprofloxacin, and gentamicin. However, these isolates exhibited resistance to the remaining antibiotics. The highest number of isolates were found resistant to cephradine (93%), followed by streptomycin (85.7%), and trimethoprim-sulfamethoxazole (71.4%). The *S*. Gallinarum isolates showed a lower resistance pattern to kanamycin and tetracycline (7.1%). *S*. Gallinarum and *S*. Pullorum both exhibited a similar pattern of antibiotic resistance. All *S*. Pullorum isolates (Fig. 2b) exhibited 100% resistance to cephradine. Additionally, *S*. Pullorum isolates showed high levels of resistance to amoxicillin (91%), trimethoprim-sulfamethoxazole (83%), and ampicillin (83%). In contrast, lower levels of resistance (16.7%) were observed for tetracycline, kanamycin, and azithromycin. Furthermore, all *S*. Pullorum isolates were susceptible (100%) to gentamicin, ciprofloxacin, and chloramphenicol.

## Discussion

In this study, the terms pre-harvest and post-harvest refer specifically to the stages of the poultry production level and the processing chain, respectively. More specifically, the pre-harvest phase encompasses the period before the birds are ready for slaughter, which includes their time on the farm and any potential exposure to environmental factors. The post-harvest phase, on the other hand, pertains to the processing and distribution of poultry products, particularly meat, after slaughter [28]. The distinction between these phases is crucial for understanding the dynamics of *S*. Pullorum and *S*. Gallinarum in both farm environments and poultry products. The time interval between these phases can influence the prevalence and antibiotic resistance patterns of these pathogens [29,30]. For example, during the pre-harvest phase, factors such as farm management practices, biosecurity measures, and bird health may contribute to the presence and resistance of SE [8]. In contrast, the post-harvest phase involves processing and handling practices that can further impact the prevalence and resistance of these pathogens in the final poultry products available to consumers [31,32].

The prevalence of these poultry pathogens was compared between the recent pre-harvest farm environmental samples (2019–2021) and the previously collected post-harvest chicken meat samples (2012–2014). We also aimed to assess the current prevalence of *S*. Gallinarum and *S*. Pullorum amidst the contemporary shift towards antibiotic-free and organic poultry farming practices. The recent pre-harvest samples align with this evolving trend. Meanwhile, the inclusion of historical post-harvest samples from 2012 to 2014 serves to establish a baseline, allowing us to discern changes or trends in bacterial prevalence over time. This temporal approach enriches our study by providing insights into the dynamic landscape of poultry farming practices and the corresponding impact on *Salmonella* prevalence. We believe this comparative study enhances the overall robustness and relevance of our investigation.

Our study has identified that pre-harvest samples collected from farm environment, particularly soils, showed frequent prevalence of SE. According to Jechalke *et al.* [33], SE is more prevalent in soil samples compared to other environmental samples in poultry farms due to its ability to survive and persist in soil by forming protective biofilms. Furthermore, the gradual accumulation of bacteria shed in faeces over time may additionally contribute to higher concentrations in the soil [34]. A study by Xie *et al.* [35] examined samples taken at a single time point from three pens across three feedlots in Texas, totaling 27 samples. The research found that the prevalence of SE was highest in soil and water, whereas faecal samples showed the lowest prevalence. At the post-harvest level, the study conducted previously by Peng *et al.* [8] observed a higher prevalence of SE collected from farmers’ markets and organic retailers compared to meat samples taken from conventional retail chicken producers. This is consistent with previous studies highlighting SE’s common and abundant presence in chickens, particularly in pasture-raised chickens [36,37]. SE is commonly found in chickens, particularly in pasture-raised settings, due to increased environmental exposure, faecal–oral transmission, interactions with wildlife, and limited biosecurity measures [38,41].

Recently, McMullin [42] reported that some poultry diseases are resurging due to the increase in free-range production. Researchers also found that both pullorum disease and fowl typhoid have re-emerged in recent years in several developing nations [43,44]. Zhou *et al*. [45] reported a thorough investigation using meta-analysis of the global prevalence of *S*. Gallinarum from 1945 to 2021 and discovered that free-range chickens had a higher prevalence of *S*. Gallinarum (24.8%) than caged chickens (7.64%). Although the prevalence of S. Gallinarum in our samples was significantly lower, the findings align with previous studies. In this study, we used LB broth with 10% sheep blood for enrichment, but more enriched media could improve the recovery of injured SE cells. That could be a potential limitation for minimum recovery. The chickens from organic retail and farmers’ markets had access to pasture areas that are often shared with other domestic animals, as a part of pasture rotation, and have likely been exposed to other wildlife. This study aimed to identify potential factors contributing to the persistence and transmission of *S*. Pullorum and *S*. Gallinarum across the poultry production and processing chain by examining and comparing their prevalence and antibiotic resistance patterns at both pre-harvest and post-harvest stages. This information is crucial for developing targeted surveillance and control strategies to mitigate the risks associated with SE contamination in poultry products [46].

In general, both *S*. Gallinarum and *S*. Pullorum cases occur sporadically. Fowl typhoid outbreaks have been documented since 2005 in commercial flocks in Belgium, Bulgaria, France, Germany, Hungary, Italy, the Netherlands, and the UK from 1 to 4 years at a time. The disease has been documented in Romania every year up to 2012, and the pathogens’ existence has been reported from 2014 to 2016 [14]. Every year, up to six backyard poultry occurrences are recorded, A significant number of commercial laying flock incidents between 2002 and 2012 were all detailed in UK reports from 2005 [47, 48]. According to WAHIS [49], the latest follow-up report on Fowl typhoid in Mexico, dated 13 April 2024, indicates an ongoing outbreak of the disease, which began on 9 February 2024. The outbreak, characterized by the recurrence of an eradicated disease, involves a total of 524 susceptible birds, resulting in five cases and two deaths. Additionally, secondary cases have been detected in San Martin Hidalgo, Jalisco, and Umán, Yucatán. Pullorum disease has been detected since 2005 in domestic flocks in the Czech Republic, Denmark, France, Germany, Greece, Hungary, Italy, the Netherlands, Norway, Poland, Romania, and the UK in single- or up to 6 year periods [50]. Up to two incidents involving pheasants and 0–3 isolations of backyard poultry each year were acknowledged in UK reports from 2011 [14]. According to WAHIS [49], the most recent pullorum disease outbreak was reported in Denmark on 20 May 2019, with the Netherlands having the previous outbreak reported on 2 July 2011, followed by Japan on 16 July 2010.

Compared to other nations, *S*. Gallinarum detection rates in developing countries have been reported in multiple studies, with 25.75% in Pakistan [20], 37.14% in Colombia [51], 69.62% in India [52], 58% in Bangladesh [53], 57.2% in Nigeria [54], and 2.4% in Malaysia [55]. Additionally, *S*. Pullorum prevalence also has been reported 82.6% in China [56], 27% in Bangladesh [53], and 6.1% in Nigeria [54]. Furthermore, the discussion raises concerns about the global prevalence of *S*. Gallinarum and *S*. Pullorum in years to come. Previous studies from different countries show varying rates of infection, indicating that this is a widespread problem that needs to be addressed at a global level. Collaborative efforts between countries can help share knowledge, best practices, and resources to combat *Salmonella* and other pathogens effectively.

Bailey and Cosby [57] found that free-range chickens in the USA exhibited a greater occurrence of *Salmonella* infection compared to those raised conventionally. This could be attributed to their increased exposure to the outdoor environment. Within the free-range production system, challenges arise in implementing biosecurity measures and mitigating environmental stressors. Free-range chickens are particularly vulnerable to various environmental stressors, such as severe weather, predation, interactions with wild birds, and intraflock aggression, surpassing the challenges encountered by chickens confined to barns or cages [58]. While it is true that *S*. Gallinarum and *S*. Pullorum are avian-specific pathogens with limited impact on humans and other animals, their significance in chicken meat samples lies in their potential implications for both animal welfare and economic sustainability in the poultry industry [59,60]. Therefore, our research not only contributes to avian health and welfare but also addresses broader issues related to consumer confidence, economic viability, and the sustainable production of poultry products. In order to ensure the general safety and quality of poultry products for consumers, this study seeks to provide important insights for the creation of efficient surveillance and control techniques by finding and comprehending the antibiotic resistance patterns of *S*. Gallinarum and *S*. Pullorum in chicken meat.

In certain nations, fowl typhoid and pullorum disease are still treated with antibiotics [61,62]. Numerous chemotherapeutic treatments have been proven to be successful in lowering mortality, but they are unable to completely eradicate the infection from a flock. Birds often retain their infection after the treatment period ends and are able to contract it again from their immediate surroundings [17]. Numerous organisms have acquired antibiotic resistance due to the indiscriminate use of antibiotics. The continued use of these medication-resistant pathogens causes the pathogen to evolve into highly resistant strains that can spread across the environment and cause future massive disease outbreaks [63]. Our study found both *S*. Gallinarum and *S*. Pullorum isolates to be highly sensitive to chloramphenicol, ciprofloxacin, and gentamicin. Consistent findings were reported in previous studies. Taddele *et al*. [64] demonstrated sensitivities of 93.3%, 88.8%, and 82% in *S*. Gallinarum isolates to gentamicin, ciprofloxacin, and chloramphenicol, respectively. Sun *et al*. [21] reported sensitivities of 100%, 99.7%, and 99.4% in *S*. Pullorum isolates to gentamicin, ciprofloxacin, and chloramphenicol, respectively. Filho *et al.* [65] noted sensitivities of 78% in *S*. Gallinarum isolates and 63% in *S*. Pullorum isolates to ciprofloxacin. *S*. Gallinarum and *S*. Pullorum isolates have frequent cephradine resistance, which has been reported on SE (specifically nontyphoidal *Salmonella* in poultry) [66,67]. The high resistance of *S*. Gallinarum and *S*. Pullorum isolates to cephradine is particularly concerning not only within the context of poultry farming but also for human health. Cephradine, belonging to the cephalosporin class, is also used in human medicine to treat various bacterial infections [68]. The observed resistance in poultry farming environments raises the risk of transmission of resistant *Salmonella* strains to humans through the food chain [69]. In cases where humans are infected with cephradine-resistant *Salmonella* strains, treatment options may be limited, leading to prolonged illness, increased healthcare costs, and potentially adverse health outcomes [70,72]. The production of extended spectrum β-lactamases is a key mechanism that grants antibiotic resistance in Salmonella. Additionally, the primary mechanisms of antibiotic resistance in *Salmonella* are the over-expression of efflux pumps and mutations in target genes (such as DNA gyrase and topoisomerase IV). However, additional processes, including alterations in the cell envelope, decreased membrane porin activity, an increase in the lipopolysaccharide (LPS) component of the outer cell membrane, quorum sensing, and biofilm formation may potentially be involved in the resistance seen in this species [73,77].

Despite the fact that the farms under this study refrain from using subtherapeutic antibiotics, it is critical to note the unexpectedly significant antibiotic resistance in *S*. Gallinarum and *S*. Pullorum. This seeming paradox can be explained by taking into account several relevant elements. One significant aspect to acknowledge is the potential influence of environmental reservoirs [78]. The antibiotic-resistant strains detected in the sampled farms might have originated from external sources, such as neighbouring farms, water reservoirs, or wildlife [28,79]. This external input could contribute to the prevalence of resistant strains within the studied farms, even in the absence of direct antibiotic application. Another key consideration is the phenomenon of horizontal gene transfer. The mechanisms facilitating the horizontal transfer of antibiotic resistance genes include transformation, conjugation transfer, transduction, membrane vesicles (MVs), and DNA packaged within virus-like particles [80]. Antibiotic resistance genes have the capability to move between different bacteria within the environment [81]. Even without the direct application of antibiotics on the sampled farms, the transfer of resistance genes between bacteria could account for the observed antibiotic resistance in *Salmonella* strains [82]. Additionally, attention should be drawn to the potential transport of resistant strains through various vectors, such as contaminated equipment, feed, or personnel. This transport mechanism could introduce antibiotic-resistant bacteria to the farms, independent of direct antibiotic administration [28,83].

Even though these biovars may not pose a direct threat to human health, their prevalence in chicken meat can lead to increased mortality rates among birds, resulting in non-profitable poultry farming [10,13]. Additionally, the growing preference among customers for antibiotic-free and organic poultry products makes it crucial to understand and monitor the presence of such pathogens in these market segments [84]. The reemergence of bacterial diseases in antibiotic-free and pasture poultry farms, as indicated by our study, raises concerns about the sustainability of these farming practices.

Moreover, the significant levels of antibiotic resistance observed within *S*. Pullorum and *S*. Gallinarum isolates accentuate the urgent need to implement judicious antibiotic practices in poultry farming. This is crucial in curbing the emergence and dissemination of antimicrobial resistance. Through the identification of antibiotic resistance patterns and prevalence in these pathogens, this study provides essential information for developing focused measures intended to reduce the occurrence of *S*. Gallinarum and *S*. Pullorum infections in poultry. This, in turn, bolsters public health and ensures the sustainability of the poultry industry.

This study demonstrates that both *S*. Pullorum and *S*. Gallinarum are present in pasture poultry farms, specifically backyard/integrated crop-livestock farms with a poultry component, which may result in higher bird mortality and economical losses. Furthermore, the contamination of post-harvest products with these pathogens underscores the necessity for enhanced processing and practices. Therefore, the implementation of regular surveillance and monitoring programmes, alongside potential mandates for future control and prevention strategies, becomes imperative in mitigating the transmission of *S*. Pullorum and *S*. Gallinarum. Lastly, the identification of specific antibiotic resistance patterns equips veterinarians and healthcare experts to prescribe suitable treatments for afflicted birds, thereby curbing the transmission of resistant strains to humans.

## supplementary material

10.1099/acmi.0.000775.v6 Uncited Fig. S1.

## Abbreviations

AHVLA: Animal Health and Veterinary Laboratories Agency
ATCC: American Type Culture Collection
BPW: buffered peptone water
CDC: Centers for Disease Control and Prevention
CFSPH: Center for Food Security and Public Health
c.f.u.: colony forming units
CLSI: Clinical Laboratory Standards Institute
FAO: Food and Agriculture Organization
ICLF: integrated crop-livestock farm
MIC: minimum inhibitory concentration
NARMS: National Antimicrobial Resistance Monitoring System
OD: optical density
OEC: observatory of economic complexity
OIE: World Organisation for Animal Health (formerly Office International des Epizooties)
PCR: polymerase chain reaction
SARE: sustainable agriculture research and education
SE: *Salmonella enterica*
*S*. Gallinarum: *Salmonella enterica* subsp. *enterica* serovar Gallinarum biovar Gallinarum
*S*. Pullorum: *Salmonella enterica* subsp*. enterica* serovar Gallinarum biovar Pullorum
USDA: United States Department of Agriculture
WAHIS: World Animal Health Information System
