# Supplementary material for: Identification of Salmonella enterica biovars Gallinarum and Pullorum and their antibiotic resistance pattern in integrated crop-livestock farms and poultry meats
Source: Access Microbiol. 2024 Sep 30;6(9):000775.v6. doi: 10.1099/acmi.0.000775.v6 (PMC11652723; doi:10.1099/acmi.0.000775.v6)
Supplement: Uncited Fig. S1. [file acmi-6-00775-s001.pdf]

Supplement Data

To verify the specificity of primers used in this study, the PCR reactions with several negative controls, specifically using water, PCR reagent (no DNA), and DNA from a highly related *Salmonella* serovar/serotype (*Salmonella enterica* serotype Typhimurium, LT2, ATCC 700720) were used along with *Salmonella* Gallinarum and *Salmonella* Pullorum (shown in Figure below). This figure confirms the primers used in this study are specific for *Salmonella* Gallinarum and *Salmonella* Pullorum.

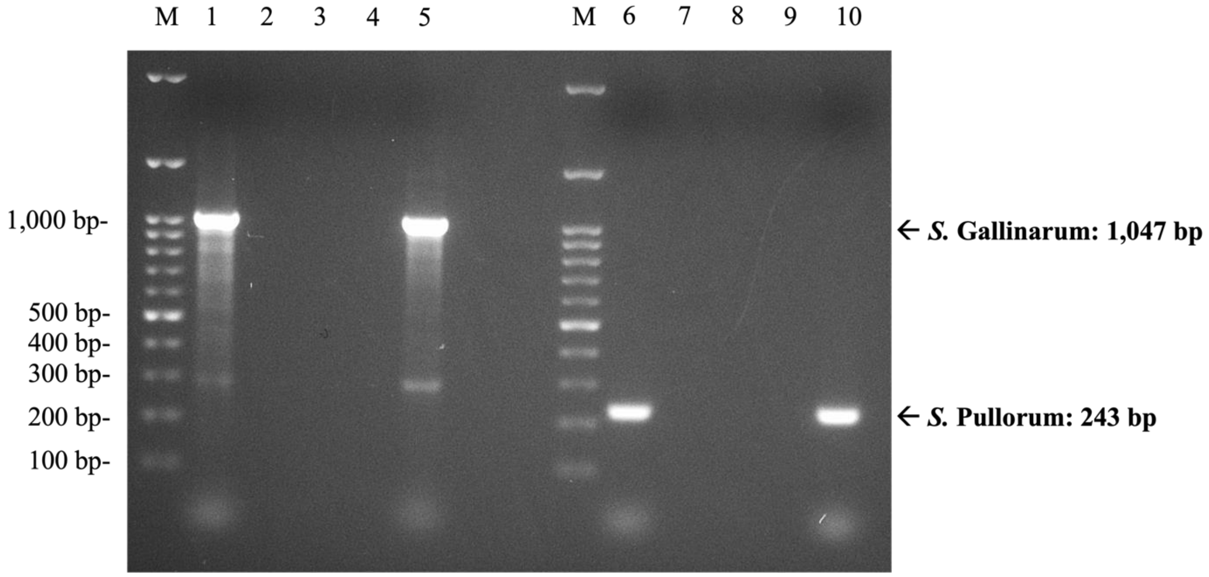

Figure: The PCR assays were performed using ratA gene to differentiate *S.* Gallinarum from *S.* Pullorum due to distinct sequence variations between these two biovars. Lane 1: *S.* Gallinarum strain collected from post-harvest samples. Lane 2: Negative control (with water). Lane 3: Negative control (PCR reagents only, no DNA). Lane 4: Negative control (with ATCC strain *S.* Typhimurium LT2). Lane 5: Positive control (with ATCC 9184 strain *S.* Gallinarum). Lane 6: *S.* Pullorum strain collected at pre-harvest samples. Lane 7: Negative control (with water). Lane 8: Negative control (PCR reagents only, no DNA). Lane 9: Negative control (with ATCC strain *S.* Typhimurium LT2). Lane 10: Positive control (with ATCC 19945 strain *S.* Pullorum).
